# The Glucagon-Like Peptide-1 Analogue Liraglutide Reduces Seizures Susceptibility, Cognition Dysfunction and Neuronal Apoptosis in a Mouse Model of Dravet Syndrome

**DOI:** 10.3389/fphar.2020.00136

**Published:** 2020-02-28

**Authors:** Shenhai Liu, Zhe Jin, Yiling Zhang, ShiKuo Rong, Wenxin He, Kuisheng Sun, Din Wan, Junming Huo, Lifei Xiao, Xinxiao Li, Na Ding, Feng Wang, Tao Sun

**Affiliations:** ^1^Ningxia Key Laboratory of Cerebrocranial Disease, Incubation Base of National Key Laboratory, Ningxia Medical University, Yinchuan, China; ^2^Department of Neurosurgery, General Hospital of Ningxia Medical University, Yinchuan, China; ^3^Department of Medical Cell Biology, Uppsala University, Uppsala, Sweden; ^4^Department of Integrated Medicine, Affiliated DongFeng Hospital, HuBei University of Medicine, Shiyan, China

**Keywords:** apoptosis, Dravet syndrome, epilepsy, GLP-1, mTOR, neuroprotection, SCN1A

## Abstract

Dravet syndrome (DS) is a refractory epilepsy typically caused by heterozygous mutations of the *Scn1a* gene, which encodes the voltage-gated sodium channel Nav1.1. Glucagon-like peptide-1 (GLP-1) analogues, effective therapeutic agents for the treatment of diabetes, have recently become attractive treatment modalities for patients with nervous system disease; however, the impact of GLP-1 analogues on DS remains unknown. This study aimed to determine the neuroprotective role of liraglutide in mouse and cell models of *Scn1a* KO-induced epilepsy. Epileptic susceptibility, behavioral changes, and behavioral seizures were assessed using electroencephalography (EEG), IntelliCage (TSE Systems, Bad Homburg, Germany), and the open field task. Morphological changes in brain tissues were observed using hematoxylin and eosin (HE) and Nissl staining. Expression of apoptosis-related proteins and the mammalian target of rapamycin (mTOR) signaling pathway were determined using immunofluorescence and western blotting in *Scn1a* KO-induced epileptic mice *in vitro*. *Scn1a* KO model cell proliferation was evaluated using the Cell Counting Kit-8 assay, and the effect of liraglutide on cellular apoptosis levels was examined using Annexin V-FITC/PI flow cytometry. Apoptotic signal proteins and mTOR were assessed using reverse transcription - quantitative polymerase chain reaction (RT-qPCR) and western blotting. Our results showed that liraglutide significantly increased mRNA ((0.31 ± 0.04) *10^-3^ vs. (1.07 ± 0.08) * 10^-3^, *P* = 0.0004) and protein (0.10 ± 0.02 *vs.* 0.27 ± 0.02, *P* = 0.0006) expression of *Scn1a* in *Scn1a* KO-induced epileptic mice. In addition, liraglutide significantly alleviated electroencephalographic seizures, the severity of responses to epileptic seizures (96.53 ± 0.45 % vs. 85.98 ± 1.24 %, *P* = 0.0003), cognitive dysfunction, and epileptic-related necrotic neurons (9.76 ± 0.91 % vs. 19.65 ± 2.64 %, *P* = 0.0005) in *Scn1a* KO-induced epileptic mice. Moreover, liraglutide protected against *Scn1a* KO-induced apoptosis, which was manifested in the phosphorylation of mTOR (KO+NS: 1.99 ± 0.31 vs. KO+Lira: 0.97 ± 0.18, *P* = 0.0004), as well as the downregulation of cleaved caspase-3 (KO+NS: 0.49 ± 0.04 vs. KO+Lira: 0.30 ± 0.01, *P* = 0.0003) and restoration of the imbalance between BAX (KO+NS: 0.90 ± 0.02 vs. KO+Lira: 0.75 ± 0.04, *P* = 0.0005) and BCL-2 (KO+NS: 0.46 ± 0.02 vs. KO+Lira: 0.61 ± 0.02, *P* = 0.0006). Collectively, these results show that liraglutide reduces seizure susceptibility and cognitive dysfunction in the mouse model of Dravet syndrome, and exerts anti-apoptotic and neuroprotective effects in *Scn1a* KO mice and cells.

## Introduction

Dravet syndrome (DS), also known as severe myoclonic epilepsy in infants, is a rare childhood epileptic encephalopathy characterized by early onset seizures, multiple types of seizures, anxiety-like behavior, severe cognitive deficits, and resistance to antiepileptic drug treatment (Brunklaus et al., 2012; Dutton et al., 2017; Knupp and Wirrell, 2018). Approximately 70–80% of DS patients have been found to have mutations in the *SCN1A* gene encoding a neuronal voltage-gated sodium channel Nav1.1 subunit and more than 90% of these *SCN1A* mutations are *de novo* (Escayg and Goldin, 2010; Brackenbury and Isom, 2011; Verbeek et al., 2011).

Glucagon-like peptide-1 (GLP-1) is generally considered as a peripheral incretin hormone that is secreted from intestinal L-cells after food ingestion. GLP-1 binds to and activates its receptor (GLP-1R) in the pancreatic islets, stimulates insulin secretion, and inhibits glucagon release in a glucose-dependent manner and improves glycemic control (Katsurada and Yada, 2016). Therefore, GLP-1 analogues such as liraglutide are currently used as a second-line therapy to treat type 2 diabetes. GLP-1 and liraglutide can pass through the blood brain barrier (BBB) (Kastin et al., 2002; McGovern et al., 2012). In addition, GLP-1 can also be synthesized by preproglucagon neurons in the brainstem including the caudal nucleus tractus solitarii (NTS) (Baggio and Drucker, 2007; Christian, 2012). The GLP-1 receptor (GLP-1R) is widely expressed in neurons of several brain subregions such as hypothalamus, hippocampus, and cortex (Cork et al., 2015; Yoshino et al., 2015), suggesting the GLP-1 signaling could modulate a variety of neuronal functions (Korol et al., 2015; Prashant et al., 2018). Indeed, it has been reported that GLP-1 analogues exert neuroprotective effects in mouse models of acute and chronic epilepsy (Koshal et al., 2018; Wang et al., 2018; Hussein et al., 2019). Previous studies have shown that different apoptosis signaling pathways including BCL-2 associated X protein (BAX)/B cell lymphoma 2 (BCL-2) proteins are involved in seizure-induced neuronal death (Guo et al., 2017; Rubio et al., 2019), and liraglutide treatment could reduce the expression of an apoptotic marker caspase-3 in the brain of a Pentylenetetrazole (PTZ)-induced kindled rat model (Hussein et al., 2019).

In addition, the mammalian target of rapamycin (mTOR) signaling pathway has attracted interest due to its critical role in regulating neuronal function, proliferation, apoptosis, and other cellular processes associated with epileptogenesis (Maiese et al., 2013; Bockaert and Marin, 2015; Citraro et al., 2016). The expression of mTOR signaling-related proteins is abnormally increased in animal models of epilepsy (Citraro et al., 2016). GLP-1 analogues can regulate mTOR expression *via* the AMP-activated protein kinase (AMPK) pathway (He et al., 2016; Zhang et al., 2017). Although several studies have shown the anticonvulsant potential of GLP-1 analogues (Koshal and Kumar, 2016; Wang et al., 2018; de Souza et al., 2019), the effect of liraglutide on the epileptogenesis and cognitive dysfunction in the DS mouse model has not been examined. In the present study, we investigated the possible anti-epileptic effect of liraglutide in *Scn1a* KO-induced epileptic mice and its potential effect on both Bcl-2 regulate apoptotic pathway and the mTOR pathway that are involved in epileptogenesis.

## Materials and Methods

### Animals

Mice with heterozygous loss-of-function mutations in *Scn1a* (*Scn1a*^+/−^) recapitulate many features of DS and provide a useful disease model. Homozygous mice (*Scn1a*^−/−^) display tremors, ataxia, seizures, and loss of righting reflex after postnatal day 9 and die by postnatal day 16. On the solely 129S6/SvEvTac strain, heterozygous mice have no overt phenotype. If mice of this 129S6/SvEvTac strain are crossed with C57BL/6, F1 heterozygotes exhibit spontaneous seizures and premature lethality (Miller et al., 2014; Kang et al., 2019). *Scn1a* KO mice created with 129S6/SvEvTac background were provided by Dr. Long-Jun Wu (Department of Cell Biology and Neuroscience, School of Arts and Sciences, Rutgers University, USA). The F1 heterozygotes (4 weeks, male) and their Wild Type (WT) littermates were used in all experiments. All of the animals were maintained in a 12-h light/dark cycle with a constant room temperature and housed in groups (five to six mice per cage) with food and water *ad libitum*. The mice were handled according to the guidelines approved by the Institutional Animal Care and Use Committee of Ningxia Medical University (IACUC Animal Use Certificate No.: SCXK [Ning] 2015-0001). All efforts were made to minimize the number of animals used and their suffering.

### Chemicals and Reagents

Liraglutide (HYP0014, purity 99.96%) was purchased from MedChemExpress (MCE, USA). Rabbit anti-SCN1A (Abcam, ab24820), p-mTOR (Abcam, ab84400), mTOR (Abcam, ab2732), Cleaved-Caspase3 (Abcam, ab2302), BAX (Abcam, ab53154), BCL-2 (Abcam, ab196495), and β-actin (Abcam, ab8227) antibodies were used as the primary antibodies based on the validation results by the manufacturers. Secondary antibodies including AlexaFluor 488-conjugated goat anti-rabbit IgG (Abcam, ab150077) were purchased from Abcam. Goat anti-rabbit IgG (LI-COR, USA) were purchased from LI-COR Bioscience (Lincoln, NE, USA).

### Genotyping the *Scn1a* KO Mice

DNA was isolated from mice tail biopsies using the TIANamp Genomic DNA kit according to the manufacturer’s instructions (Tiangen Biotech, Beijing, China). The DNA concentration was determined using a spectrophotometer (Nanodrop 2000; Thermo Fisher Scientific, Waltham, MA, USA). The presence of the *Slcn1a* exon 1 deletion was identified by the standard polymerase chain reaction (PCR) technique using the enzyme Hot Start High-Fidelity DNA Polymerase (New England Biolabs, Ipswich, MA, USA) and specific primers. The sequences of the primers used for mouse genotyping were as follows: common primer 5′-AGTCTGTACCAGGCAGAACTTG-3′, wild type reverse primer 5′-CCCTGAGATGTGGGTGAATAG-3′, mutant reverse prime 5′-AGACTGCCTTGGGAAAAGCG-3′. The PCR reaction was performed in a mixture containing 2.5 μl common primer and 1.25 μl wild type reverse primer and 1.25 μl mutant reverse primer, 3 μl nuclease-free water, 12 μl DNA polymerase, and 5 μl DNA. After the initial denaturation for 30 s at 98°C, 35 PCR cycles were performed (98°C for 10 s, 56°C for 30 s, and 72°C for 2 min), followed by a final extension at 66.5°C for 2 min. The PCR products were observed after the electrophoresis on a 2% agarose gel stained with GelRed (Biotium, Fremont, CA, USA) and loaded with a 700bp DNA ladder (Biomed, Beijing, China). The genotype results were confirmed by sequencing of the PCR products (Sangon Biotech Co., Ltd., Shanghai, China).

### Animal Experimental Protocol

A total of 120 WT mice and F1 heterozygous *Scn1a* KO mice were randomly divided into five groups: (I) Liraglutide treated WT group (WT + Lira, N = 24), which received liraglutide treatment (150 μg/kg, i.p) once daily for 14 days (During et al., 2003; Yoshino et al., 2015). (II) Normal saline treated WT group (WT + NS, N = 24), which received the same volume of 0.9% normal saline once daily for 14 days. (III) Normal saline treated F1 heterozygous *Scn1a* KO mice group (KO + NS, N = 24). (IV) Liraglutide treated F1 heterozygous *Scn1a* KO mice group (KO + Lira, N = 24). (V) Valproic acid treated F1 heterozygous *Scn1a* KO mice group (KO + VPA, N = 24), which received the same volume of valproic acid once daily for 14 days. The time point at which the mice were sacrificed to take out brain tissues was consistent with the liraglutide administration time (**Supplementary Figure 1**).

### Blood Glucose Measurements

We collected blood samples from the tail vein and measured the fasting blood glucose. Blood glucose levels were measured by the Sannuo blood glucose meter (Sinocare Inc. China) before treatment and before sacrifice at different time points (12 h, 1 d, 3 d, 7 d, 14 d after the liraglutide administration). The measurements were repeated three times to reduce errors and the average value was calculated as the blood glucose level.

### Electroencephalography (EEG) Measurement

Mice were anesthetized with 1% pentobarbital (40 mg/kg, i.p) and maintained at normal body temperature on a feedback-controlled heating blanket (TR-200, Safebio, Shanghai, China). The mice were then transferred to a stereotactic frame and a midline scalp incision was made to expose the skull. EEG electrodes were skull-mounted on the left amygdala (coordinates from the bregma: AP = −1.2 mm and L = −9 mm) and the frontal cortex (coordinate from the bregma: AP = 2 mm and L = −2 mm). Behavioral seizures and EEG were observed and recorded by a biomedical signal acquisition and processing system (BL-420 N, Techman Software, Chengdu, China). A novel definition of High-Voltage Sharp Waves (HVSWs) was used: sharp waves with a high amplitude at least three times the EEG baseline, a duration of at least 5 s, and a frequency of at least 2 Hz (Twele et al., 2016).

### Evaluation of Behavioral Seizures

The severity of seizures was assessed and classified according to the Racine stage (Racine, 1972): stage 0: normal behavior; stage 1: mouth and facial movement; stage 2: head nodding; stage 3: forelimb clonus; stage 4: rearing with forelimb clonus; and stage 5: rearing and falling with forelimb clonus. In addition, stage 5 seizures had a definite termination before onset, and status epilepticus was defined as the duration of stage 5 seizures unless intentionally stopped. First abnormal behavior was referred to facial automatism and excessive salivation. The severity of seizures can be evaluated using the sum score of each mouse and the duration of each experiment according to the following formula: Seizure severity = Σ (total scores of one specified mouse)/time of experiment.

### IntelliCage

IntelliCage system program settings and experimental protocol: (1) Adaptation and Free exploratory stage: All mice could move freely in the cage, the valves in the four corners were open, and the mice were free to choose the corner to drink water. The number of visiting and nosepoke in each corner of each mouse was recorded to assess the animal’s cognitive ability in the new environment, which was set to 5 days. (2) Nosepoke learning stage: At this stage, the program was set to the four corners where the valves were closed and the mouse must learn to open the valve to drink water. After the nosepoke learning, the valve will be closed after the end of the visiting. This stage was set to 5 days. (3) Position learning stage: The corner with the least number in the nosepoke learning stage was defined as the “correct” corner, and the remaining three corners were defined as the “wrong” corners. All mice can only open and drink water when they nosepoke the corner that was defined as “correct”. The error visit rate of each mouse was recorded to evaluate the position learning ability, which was set to 7 days. (4) Reversal position learning stage: The corner corresponding to the diagonal of the “correct” corner in the position learning stage was defined as the “correct” corner, and the remaining corners were defined as the “wrong” corners. The error visit rate of each mouse was also recorded to evaluate the reversal position learning ability, which was set to 7 days.

### The Open Field Task

Mice were placed in a closed planar area (length: 50 cm; width: 50 cm; depth: 40 cm) for 10 min and mice activity was recorded using Smart 3.0 video tracking software (Panlab, Spain). A 50 cm (length) × 50 cm (width) planar area was digitally divided into 20 quadrants of the same size (6 central quadrants and 14 peripheral quadrants). The 6 central quadrants are collectively referred to as the central region, and the 16 peripheral quadrants are collectively referred to as the peripheral regions. The system automatically recorded the travel distance (cm) and the time (s) that mice spent in the central area and the surrounding area, and analyzed the data.

### Immunofluorescence Staining

Mice were anesthetized in 1% pentobarbital (40 mg/kg, i.p) and perfused with saline through the heart, then followed by 4% paraformaldehyde. After the decapitation, the mouse brain was quickly removed, and post fixed in 4% paraformaldehyde for 15 h at room temperature (RT). The brains were soaked in the 30% sucrose solution for cryopreservation for 24 h. Brain tissues were cut using a cryostat (Leica, Wetzlar, Germany) and 30-μm-thick sections were collected. Non-specific binding was blocked for 1 h at RT using 3% normal goat serum and 0.1% Triton-X-100 in phosphate-buffered saline (PBS). Rabbit anti-SCN1A (1:500), p-mTOR (1:500), mTOR (1:500), Cleaved-Caspase3 (1:500), BAX (1:500), and BCL-2 (1:500) antibodies were used as the primary antibodies based on the validation results from the manufacturers. After washing in PBS 3 times, the sections were incubated with the secondary antibody AlexaFluor 488-conjugated goat anti-rabbit IgG (1:500) for 1 h at RT. The sections were then washed, counterstained with 4,6-diamidino-2-phenylindole (DAPI) (ZLI-9557, ZSGB-BIO, Beijing, China), and covered with a coverslip. The images were captured with a Leica DM6 fluorescence microscope (Leica, Germany). Image-pro plus 6.0 software (Media Cybernetics, Bethesda, MD, USA) was used for quantification of the average optical density (AOD) of images.

### Hematoxylin and Eosin Staining

Hematoxylin & Eosin (HE) staining was conducted according to a conventional protocol. Briefly, 30-μm-thick sections were stained with hematoxylin solution for 5 min and then immersed five times in 1% acidic ethanol (1% HCl in 70% ethanol) followed by rinsing in distilled water. The sections were further stained with eosin solution for 3 min, then dehydrated with a gradient alcohol and cleared in xylene. The mounted slides were examined and photographed using a Leica DM6 fluorescence microscope (Leica, Germany).

### Nissl Staining

The rehydrated mouse cortex sections were stained in a cresyl violet solution at 56°C for 1 h and then washed with deionized water. The sections were further maintained in the Nissl differentiation solution for at least 2 min until a colorless background was observed under the microscope, dehydrated (each in 50, 60, 70, 80, 95, and 100% ethanol for 3 min), washed in xylene, and fixed with neutral balsam. The sections were photographed using a Leica DM6 fluorescence microscope (Leica, Germany) and cells in the region of interest from the Nissl stained image were counted with Image-pro plus 6.0 software (Media Cybernetics, Bethesda, MD, USA).

### *Scn1a*-Knockout Cell Line

The mouse hippocampal neuronal cell line (HT22) with the knockout of *Scn1a* was constructed and stored in the liquid nitrogen at the Ningxia Key Laboratory of Cerebrocranial Diseases (Shi et al., 2019). The revived cells were passaged with a 0.25% trypsin-EDTA solution (Solarbio, China). The cells were maintained in dulbecco’s modified eagle medium (DMEM) (Bioind, USA) containing 10% fetal bovine serum (FBS) (Bioind, USA) and 1% penicillin-streptomycin (Solarbio, China) in an incubator with 5% CO_2_ at 37°C.

The cells were divided into four experimental groups: (I) HT22 cell, medium-treated group (HT22 Control), (II) HT22 cell, liraglutide-treated group (HT22 + Lira), (III) *Scn1a* KO HT22 cell, medium-treated group (KO Control), (IV) *Scn1a* KO HT22 cell, liraglutide-treated group (KO + Lira). Subsequently, the cells were seeded into a 96-well plate at a density of 1×10^5^ cells/well, and then liraglutide at a concentration of 8, 10, or 12 nM was added to the cells. The cells were further cultured in the incubator, and then collected at 24, 48 and 72 h. An inverted phase contrast microscope (Leica, Germany) and cell counter (Bio-RAD, USA) were used to observe and measure the cell numbers under different conditions.

### Cell Proliferation Assay

The Cell Counting Kit-8 assay (Dojindo, Japan) was used to evaluate the cell proliferation. Briefly, at 24, 48, and 72 h, the *Scn1a* knockout HT22 cells were treated with different concentrations of liraglutide and cultured in the 96-well plate at a density of 1×10^4^ cells/well. The blank wells with the culture medium were used for the background detection, and three duplicate wells were set for each group. After each time point of harvest and washing once with PBS, Cell Counting Kit-8 solution (10%) was added to individual wells, and the plate was incubated at 36°C for 1 h. Absorbance at 450 nm was measured using a microplate reader (Bio-τeck, USA). The measurement was repeated and the optical density (OD) values were averaged. The cell proliferation rate was calculated using the formula: Cell proliferation rate (%) = ((treated cell OD)) − (blank OD))/((control group OD) − (blank OD)) × 100.

### Flow Cytometry

Flow cytometry was used to assess the effect of liraglutide on the apoptosis of *Scn1a* knockout HT22 cells. Annexin V-FITC/Propidium Iodide (PI) double staining cell apoptosis detection kit was purchased from BestBio (BestBio, Shanghai, China). The cells were seeded in the 96-well plate at a density of 1×10^5^ cells/well. Each group consisted of three duplicate wells and cultured for 48 h. The cells were detached by EDTA- trypsin and centrifuged at 2,000 rpm for 5 min, and washed twice with PBS and 500 μl of binding buffer. AnnexinV-FITC and PI were added to the cells and further incubated at RT for 15 min in the dark. The apoptosis rate of each group was analyzed by Cytoflex flow cytometer (BECKMAN COULTER) when the number of cells was up to 1 × 10^4/^tube.

### Reverse Transcription-Quantitative Polymerase Chain Reaction (RT-qPCR)

The total RNA was extracted from cells or brain tissues using the RNA extraction kit manufactured by Omega according to the manufacturer’s protocol. First-strand cDNA was generated using reverse transcription kit (RR036A) manufactured by TAKARA. First-strand cDNA samples were used as the PCR templates with gene-specific forward and reverse primers (**Supplementary Table 1**, primers synthesized by Shanghai Shenggong Bioengineering Co., Ltd). The cDNA was amplified using qPCR kit (RR820A, TAKARA). The PCR parameters were set as follows: denaturation at 95°C for 2 min, followed by 40 cycles of denaturation at 95°C for 10 s, annealing at 58°C for 30 s, and extension at 72°C for 30 s. After the PCR amplification, data analysis was performed using Bio-Rad IQ5 software and *Gapdh* was used as a reference gene. The relative quantification (2^-△Ct^ or 2^-△△Ct^) method was used to present the data. All experiments were performed in triplicates.

### Western Blotting Assay

Total proteins from the brains and *Scn1a* KO HT22 cells were prepared and extracted using the BCA Protein Extraction Kit (KGP2100, KeyGEN, Nanjing, China). Protein concentration was measured using the BCA Protein Assay Kit (KGP902, KeyGEN, Nanjing, China). Equal amounts of protein (50 μg per lane) were resolved on 8 or 10% sodium dodecyl sulfate (SDS) - polyacrylamide gel (SDS-PAGE), and then transferred onto 0.22 μm polyvinylidene fluoride (PVDF) membrane (Millipore, USA). When the protein transfer was completed, membranes were blocked with 5% non-fat milk for 1 h, followed by incubation overnight at 4°C with rabbit anti-SCN1A (1:500), p-mTOR (1:500), mTOR (1:500), Cleaved-Caspase3 (1:500), BAX (1:500), BCL-2 (1:500), or β-actin (1:1,000) primary antibody based on the validation results from the manufacturers. After incubation with primary antibody, membranes were washed with TBST three times for 5 min each time. β-actin served as internal references. Membranes were further incubated with the corresponding secondary antibody goat anti-rabbit IgG (1:1,000; LI-COR, USA) for 1.5 h. Quantification of bands was performed from optical density values using the Odyssey CLX instrument system (LI-COR, USA). All experiments were performed in triplicates.

### Statistical Analysis

Statistical analysis was performed using PRISM 6 (GraphPad Software, California, USA). The data were presented as mean ± standard deviation (SD). Two-group comparisons were evaluated with two-tailed Student’s *t* test. To compare three or more groups, a one-way or two-way ANOVA followed by a Dunnett, Newman-Keuls, or Bonferroni *post hoc* test was used. *P* < 0.05 was considered statistically significant. At least three independent experiments were performed for each condition.

## Results

### Genotype Verification of *Scn1a* KO Mice and the Increase of Scn1a Expression in the Brain of *Scn1a* KO Mice After Liraglutide Treatment

**Figure 1:**
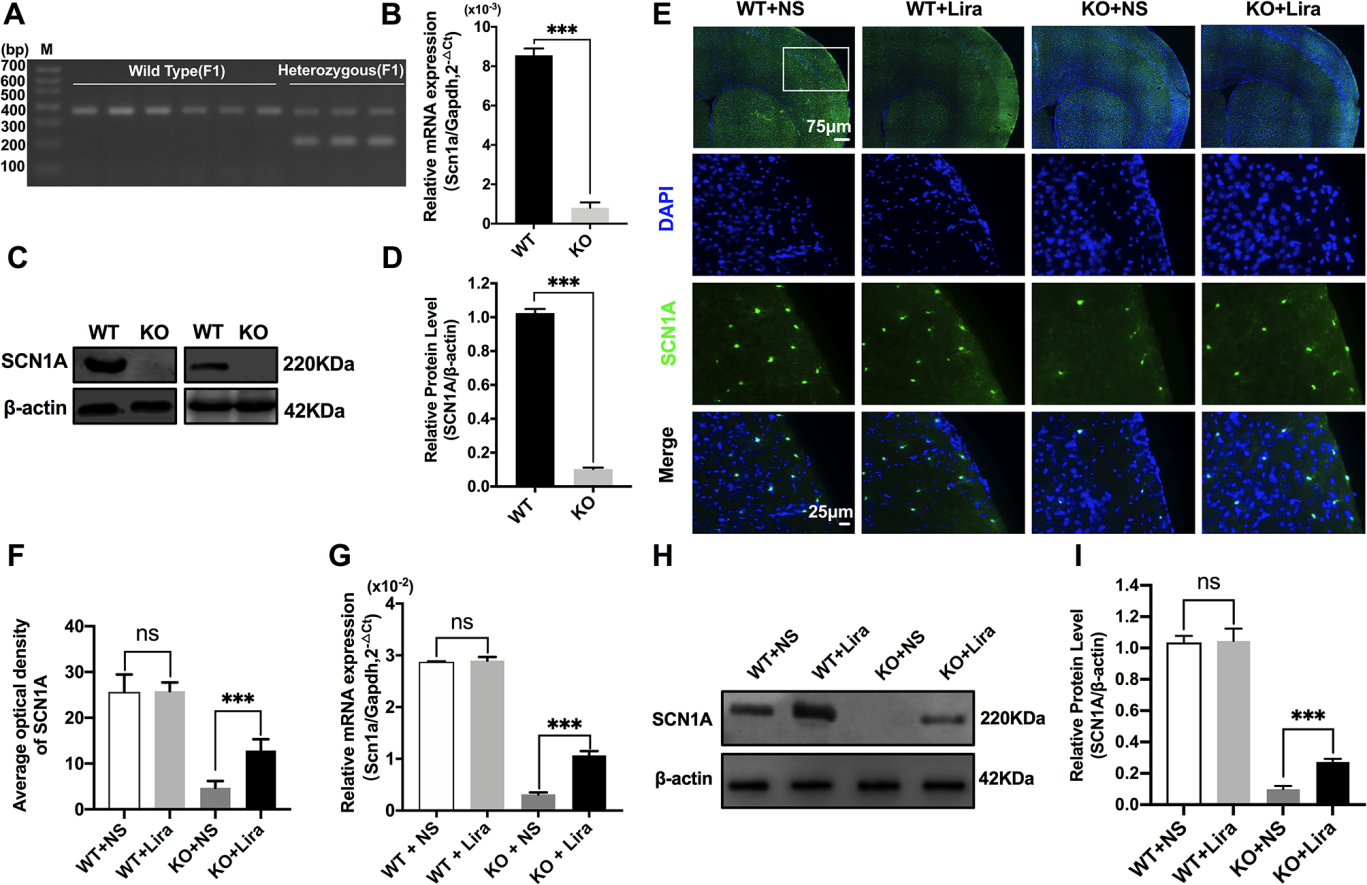
Phenotype verification of *Scn1a* KO mice and the increase of SCN1A expression after liraglutide treatment in the brain of *Scn1a* KO mice. **(A)** PCR was used to identify wild-type and *Scn1a* KO heterozygous (F1) genotypes. (M: DNA marker). **(B)** RT-qPCR analysis of the *Scn1a* mRNA expression from brains of wild-type (WT) and *Scn1a* KO heterozygote (KO) mice (N = 24 mice per group; *Student’s t-test*, *P* < 0.001). **(C)** Representative western blotting showing SCN1A protein expression in brains of WT and *Scn1a* KO mice. **(D)** Summary graph of western blotting analysis demonstrating a significant reduction in the SCN1A protein in brains of *Scn1a* KO as compared to WT mice (N = 24 mice per group; *Student’s t-test*, *P* < 0.001). **(E)** Representative images of immunofluorescent staining with SCN1A (green), and DAPI (blue) in the ipsilateral side of the cortex of the WT and *Scn1a* KO mice. Scale bar: 75 μm for the full-scale images and 25 μm for the magnified images. **(F)** Summary graph of the average optical density of SCN1A fluorescent staining measured in images **(E)** demonstrating a profound increase of SCN1A in the cortex of *Scn1a* KO mice after liraglutide administration (N = 24 mice per group; *Student’s t-test, P* < 0.001). **(G)** RT-qPCR analysis of the SCN1A mRNA expression after liraglutide administration in brains of WT and *Scn1a* KO mice (N = 24 mice per group; *One-way ANOVA, Student’s t-test*). **(H)** Representative western blotting showing SCN1A protein expression in brains of the WT and *Scn1a* KO mice. **(I)** Summary graph of western blotting analysis demonstrating a dramatic increase in the SCN1A protein from brains of *Scn1a* KO after liraglutide administration (N = 24 mice per group; *One-way ANOVA, Student’s t-test*). The relative expression of SCN1A was normalized to reference controls *Gapdh* and β-actin in RT-qPCR and Western blotting, respectively. Data were presented as mean ± SD; ***, *P* < 0.001; ns, not significant. All experiments were performed in triplicate. WT, Wild-type; KO, *Scn1a* Knockout heterozygotes (F1); NS, Normal saline; Lira, Liraglutide.

Heterozygous mice (*Scn1a*^+/−^, 129S6/SvEvTac strain) were crossed with WT mice (C57BL/6 strain), and F1 generation mice were genotyped. Based on the exon 1 sequence of mouse *Scn1a* gene, a common primer, WT reverse primer, and mutant reverse primer were designed to amplify DNA fragments using PCR. Exon 1 of the mouse *Scn1a* gene was replaced by a neomycin resistance cassette; as such, the expected PCR products were 357 bp for WT mice, and 200 bp and 357 bp for heterozygous mice (**Figure 1A**). According to the genotyping results, the successful generation of *Scn1a* KO animals (F1 generation) was achieved. The F1 heterozygotes (4 weeks, male) and their Wild Type (WT) littermates were used in all experiments. RT-qPCR, western blotting (WB), and immunofluorescence staining were used to further assess the effect of replacing exon 1 of the mouse *Scn1a* gene with a neomycin resistance cassette on Scn1a expression in the mouse brain. As shown in **Figure 1B**, WT and *Scn1a* KO littermates exhibited clearly different *Scn1a* mRNA expression in the brain. Statistical analysis revealed a significant decrease in the level of *Scn1a* mRNA expression in the *Scn1a* KO group compared with the WT group (WT: (8.55 ± 0.35) *10^-3^ vs. (0.81 ± 0.27) * 10^-3^, *P* = 0.0001, **Figure 1B**). In addition, SCN1A protein expression levels in age-matched littermates were detected using WB with whole-cell protein extracts from the brain. As expected, *Scn1a* KO mice demonstrated no evidence of SCN1A protein production, as the band corresponding to SCN1A was almost totally absent (WT: 1.02 ± 0.02, KO: 0.10 ± 0.01, *P* < 0.001, **Figures 1C, D**).

The specific distribution of SCN1A in the mouse brain was also examined by immunofluorescence staining. Strong SCN1A-like immunoreactivity was observed in neurons, mostly in the cortex and hippocampus (**Figure 1E**). Interestingly, compared with *Scn1a* KO mice that were given normal saline, the expression of SCN1A was increased in the same brain region in *Scn1a* KO mice after Lira administration (KO+NS: 4.73 ± 1.46, KO+Lira: 12.85 ± 2.48*, P* < 0.001, **Figures 1E, F**). RT-qPCR and WB were used to further verify this result. Both Scn1a mRNA and protein levels in the brain of *Scn1a* KO mice were significantly increased after Lira administration (mRNA, KO+NS: (0.31 ± 0.04) *10^-2^, KO+Lira : (1.07 ± 0.08) * 10^-2^, **Figure 1G**; protein, KO+NS: 0.10 ± 0.02, KO+Lira: 0.27 ± 0.02*, P* < 0.001, **Figures 1H, I**). In contrast, lira administration did not change the mRNA and protein level in the brain of WT mice (**Figures 1E–I**). Lira had no effects on blood glucose levels in either WT or *Scn1a* KO mice that were tested before euthanization at different time points (**Supplementary Figure 2**).

### Liraglutide Reduces Seizure Susceptibility and Cognitive Dysfunction in *Scn1a* KO-Induced Epileptic Mice

#### Behavioral Seizures and EEG Recording

**Figure 2:**
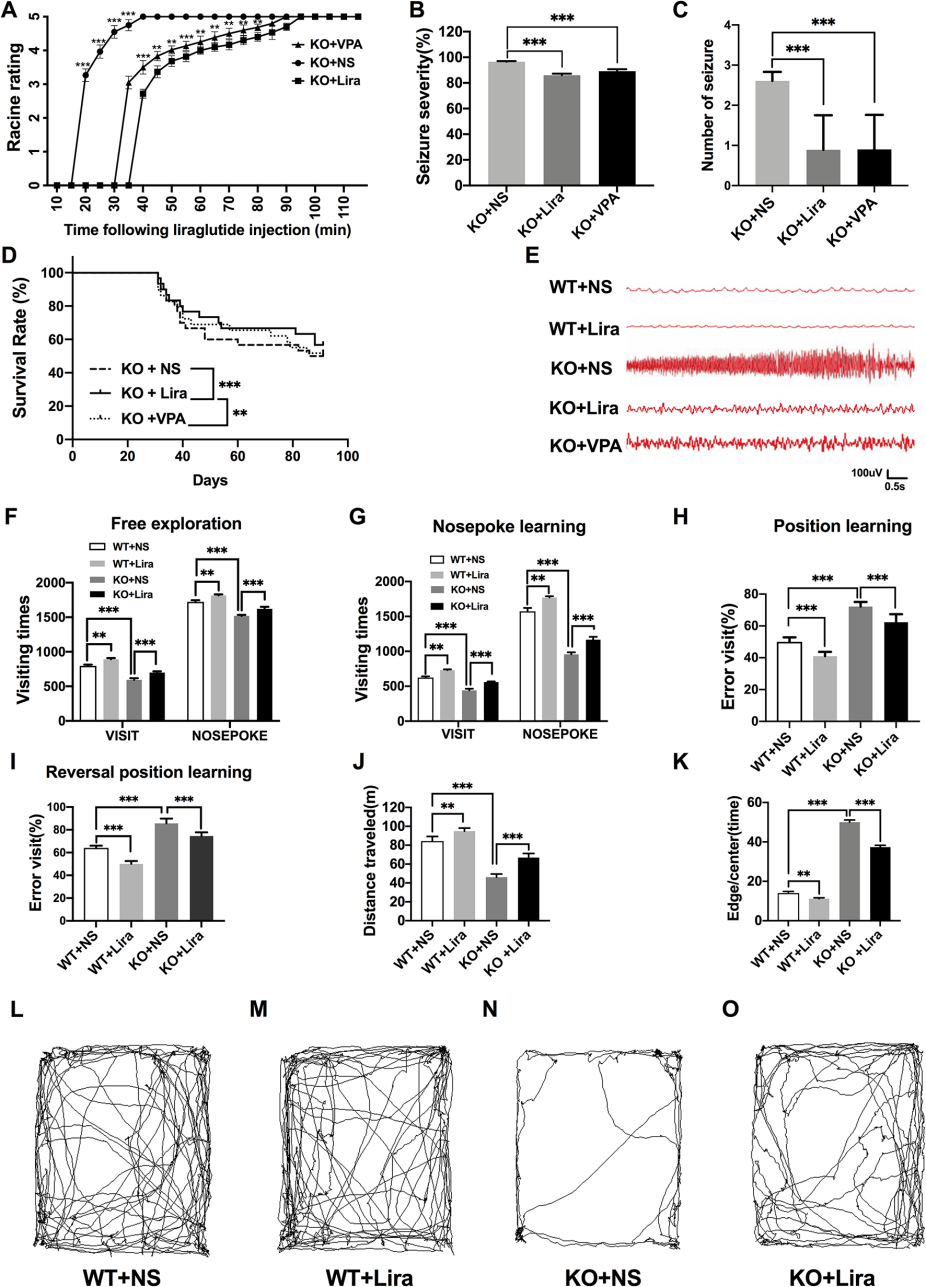
Effects of liraglutide on behavioral performance in *Scn1a* KO-induced epileptic mice. **(A)** Behavioral seizure progression on the racine scale (N = 24 mice per group; *Two-way ANOVA*). **(B)** The total seizure severity evaluation (N = 24 mice per group; *Student’s t-test*, *P* < 0.001). **(C)** The number of seizures daily in each group over the duration of the experiment (N = 24 mice per group; *Student’s t-test*, *P* < 0.001). **(D)** Survival curves showing a statistically significant difference in survival in each group over the duration of the experiment (N = 24 mice per group; *One-way ANOVA, Student’s t-test*). **(E)** A representative EEG recording of seizure activity in the WT and *Scn1a* KO mice. **(F)** Free exploratory (N = 24 mice per group; *Two-way ANOVA, Student’s t-test*). **(G)** Number of visits and nosepoke in nosepoke learning (N = 24 mice per group; *Two-way ANOVA, Student’s t-test*). **(H)** Number of correct visits in position learning (N = 24 mice per group; *One-way ANOVA, Student’s t-test*). **(I)** Number of correct visits in reversal position learning (N = 24 mice per group; *One-way ANOVA, Student’s t-test*). **(J)** Significant impairment was found in total distanced traveled (N = 24 mice per group; *One-way ANOVA, Student’s t-test*). **(K)** Significant impairment was found in total Edge/Centre time (N = 24 mice per group; *One-way ANOVA, Student’s t-test*). **(L–O)** Difference in thigmotaxis behavior between NS and Lira in WT or *Scn1a* KO mice was found, respectively, which was shown in a computer-generated trace of the animal’s movements over 10 min. Data were presented as mean ± SD; **, *** represent *P* < 0.01, and *P* < 0.001, respectively. All experiments were performed in triplicate. WT, Wild-type; KO, *Scn1a* Knockout heterozygotes (F1); NS, Normal saline; Lira, Liraglutide.

The functional significance of Lira in the susceptibility to *Scn1a* KO induced-epileptic seizures was investigated. Seizure behavior was evaluated and compared between WT and *Scn1a* KO mice after Lira administration. Seizure stage scores were used for latency and duration of seizures to assess epilepsy susceptibility. As shown in **Figure 2A**, *Scn1a* KO mice exhibited seizure behavior for more than 2 h and exhibited severe tonic-clonic seizures before status epileptic seizures to gradually reach stage 5 seizures. However, the duration of seizure behavior in *Scn1a* KO mice after Lira injection was prolonged (*P* < 0.001; **Figure 2A**) and the duration of seizures was reduced (*P* < 0.001; **Figure 2A**), followed by seizures that appeared to be relatively mild. Compared with the Lira group, the duration of seizure behavior was reduced and the Racine rating was higher in VPA group (*P* < 0.001; **Figure 2A**). Both lira and VPA administration markedly reduced seizure severity in *Scn1a* KO mice compared to NS control mice (KO+NS: 96.53 ± 0.45, KO+Lira: 85.98 ± 1.24, KO+VPA: 89.12 ± 1.61*, P* < 0.001, **Figure 2B**). As shown in **Figure 2C**, the number of seizures was significantly reduced in both Lira and VPA group (KO+NS: 2.63 ± 0.84, KO+Lira: 1.01 ± 1.15; KO+VPA: 1.02 ± 1.19*, P* < 0.001, **Figure 2C**). Survival curves showed that Lira markedly reduced the mortality compared with *Scn1a* KO NS control group and VPA group (**Figure 2D**).

In addition, cortical electrodes were implanted to measure the EEG seizure activity in mice. As shown in **Figure 2E**, *Scn1a* KO control mice (KO+NS) exhibited a higher frequency and magnitude of electroencephalographic seizure activity compared with WT mice. The administration of Lira or VPA markedly reduced the frequency and amplitude of EEG seizure activity (**Figure 2E**).

#### Cognitive Performance

We evaluated the cognitive performance of WT and *Scn1a* KO induced epileptic mice before and after the Lira administration with the IntelliCage system (adaptation and free exploration test, nose poke learning stage test, position learning stage test, and reversal position learning stage test) and smart 3.0 mouse behavioral system (the open field task).

Compared with WT mice, *Scn1a* KO mice exhibited significantly shorter visiting time (WT+NS: 796.33 ± 17.67, KO+NS: 593.83 ± 22.99, *P* < 0.001, **Figure 2F**) and the number of nose pokes was significantly reduced in the adaptation and free exploration test (WT+NS: 1722.83 ± 23.12, KO+NS: 1519.33 ± 15.18, *P* < 0.001, **Figure 2F**). The administration of Lira significantly increased visiting times (WT+NS: 796.33 ± 17.67, WT+Lira: 892.83 ± 16.51, *P* < 0.01, **Figure 2F**; KO+NS: 593.83 ± 22.99, KO+Lira: 699.33 ± 18.96, *P* < 0.001, **Figure 2F**) and the number of nose pokes in both WT and *Scn1a* KO mice (WT+NS: 1722.83 ± 23.12, WT+Lira, 1815.83 ± 14.85, *P* < 0.01, **Figure 2F**; KO+NS: 1519.33 ± 15.18, KO+Lira: 1621.01 ± 30.38, *P* < 0.001, **Figure 2F**).

In the nose poke learning stage test, *Scn1a* KO mice had significantly shorter visiting times (WT+NS: 625.01 ± 16.09, KO+NS: 441.16 ± 22.67, *P* < 0.001, **Figure 2G**) and the number of nose pokes was significantly reduced (WT+NS: 1572.51 ± 48.78, KO+NS: 956.67± 29.92, *P* < 0.001, **Figure 2G**), as compared with WT mice. Compared with *Scn1a* KO mice, the Lira group had significantly longer visiting times (WT+NS: 625.01 ± 16.09, WT+Lira, 730.33 ± 11.07, *P* < 0.01, **Figure 2G**; KO+NS: 441.16 ± 22.67, KO+Lira, 560.01 ± 9.19, *P* < 0.001, **Figure 2G**), and the number of nose pokes was significantly increased (WT+NS: 1572.51 ± 48.78, WT+Lira: 1770.67 ± 19.02, *P* < 0.01, **Figure 2G**; KO+NS: 956.67± 29.92, KO+Lira: 1166.83 ± 41.57, *P* < 0.001, **Figure 2G**).

In both position and reversal position learning stage tests (**Figures 2H, I**), *Scn1a* KO mice had significantly higher error visiting rate compared with WT mice (position test, WT+NS: 50.01 ± 2.82, KO+NS: 72.17 ± 2.93, *P* < 0.001; reversal position test, WT+NS: 64.02 ± 2.01, KO+NS: 85.67 ± 4.18, *P* < 0.001). Lira administration significantly decreased error visiting rates in WT and *Scn1a* KO mice in both tests (position test, WT+NS: 50.01 ± 2.82, WT+Lira: 41.01 ± 2.76, *P* < 0.001; reversal position test, KO+NS: 85.67 ± 4.18, KO+Lira: 74.5 ± 3.27, *P* < 0.001; **Figure 2I**).

We further investigated the cognition-related behavior of WT and *Scn1a* KO mice administered with NS or Lira using the open field task experiment. Compared with WT mice, the distance traveled by *Scn1a* KO mice decreased significantly (WT+NS: 84.33 ± 4.93, KO+NS: 46.01 ± 3.46, *P* < 0.001, **Figures 2 J, L, N**), and the residence time in the central region was significantly prolonged (WT+NS: 14.01 ± 2.09, KO+NS: 50.01 ± 2.83, *P* < 0.001, **Figures 2K, L, N**). Lira treatment significantly increased traveled the distance (WT+NS: 84.33 ± 4.93, WT+Lira: 94.83 ± 3.31, *P* < 0.01; KO+NS: 46.01 ± 3.46, KO+Lira: 66.83 ± 4.41, *P* < 0.001; **Figures 2J–O**), and prolonged the residence time in the edge region (WT+NS: 14.01 ± 2.09, WT+Lira: 11.17 ± 1.17, *P* < 0.01; KO+NS: 50.01 ± 2.83, KO+Lira: 37.33 ± 2.25, *P* < 0.001; **Figures 2K–O**) in both WT and *Scn1a* KO mice.

Collectively, these results suggest that *Scn1a* KO deficiency significantly aggravated the susceptibility and severity of seizures and exacerbated cognitive dysfunction.

### Liraglutide Alleviates Neuronal Damage Following *Scn1a* KO-Induced Status Epilepticus

**Figure 3:**
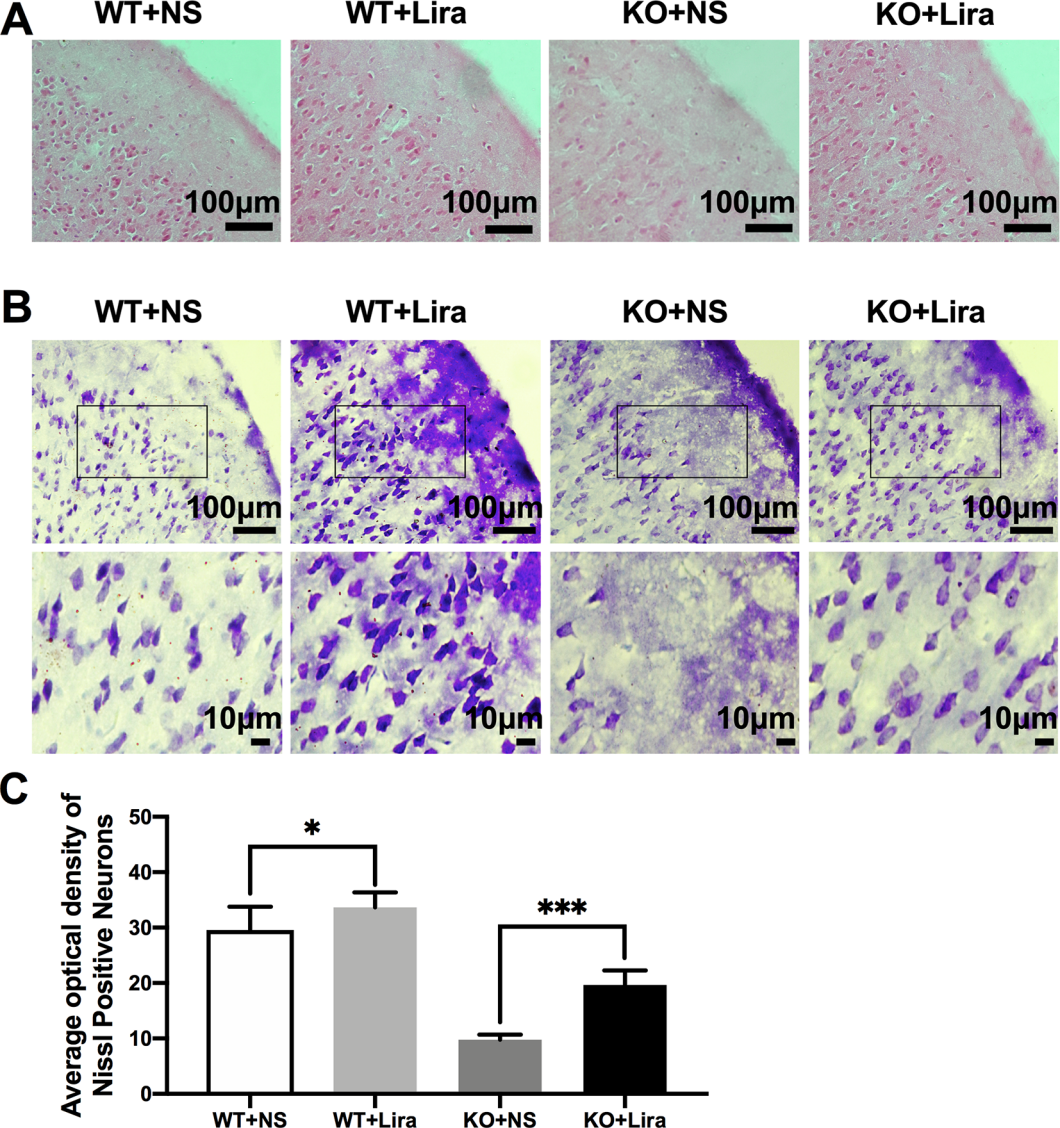
HE staining and Nissl staining showed neuron damage in the cortex. **(A)** Representative images of HE staining of the cortex. Scale bar: 100 μm. **(B)** Representative images of Nissl staining of the cortex. Scale bar: 100 and 10 μm. **(C)** Histograms of the Nissl-positive neurons obtained *via* Nissl staining in the cortex (N = 24 mice per group; *One-way ANOVA, Student’s t-test*). Data were presented as mean ± SD; *, *** represent *P* < 0.05, and *P* < 0.001, respectively. All experiments were performed in triplicate. WT, Wild-type; KO, *Scn1a* Knockout heterozygotes (F1); NS, Normal saline; Lira, Liraglutide.

To investigate *Scn1a* KO-induced neuron damage and whether this damage was alleviated by administration of Lira, the lesion conditions of the cortex after 14 days following *Scn1a* KO-induced epilepsy were analyzed using HE and Nissl staining. Compared with the WT group (WT+NS, **Figure 3A**), the HE staining showed that cortical necrosis in the *Scn1a* KO group (KO+NS, **Figure 3A**) was severe, and the cells were disorderly arranged with unclear edges. Nucleus pyknosis, cytoplasmic staining, and cell body shrinkage were observed in cortical cells of the *Scn1a* KO mice (KO+NS, **Figure 3A**). In contrast, cortical cells in the *Scn1a* KO mice treated with Lira were neatly arranged with and displayed relatively normal nuclear morphology (KO+Lira, **Figure 3A**). The number of necrotic neurons was also markedly reduced (KO+Lira, **Figure 3A**). The Nissl staining results were consistent with those of the HE staining. Compared with the WT mice, the apparent neuron loss featured as loose, widened, and diffuse cell layers was observed in similar cortex brain regions of the *Scn1a* KO mice (KO+NS, **Figure 3B**). After the Lira treatment, the neuron loss was significantly reduced in similar cortex brain regions of the *Scn1a* KO mice (KO+NS: 9.76 ± 0.91, KO+Lira: 19.65 ± 2.64, *P* < 0.001, **Figures 3B, C**). These results suggest that *Scn1a* KO deficient mice exhibit varying degrees of aggravated neuronal lesions in the cortex. However, Lira intervention exerts a neuroprotective effect to alleviate this neuronal damage.

### Liraglutide Inhibits Phosphorylation mTOR Hyperactivation in the Brain of *Scn1a* KO-Induced Epileptic Mice

**Figure 4:**
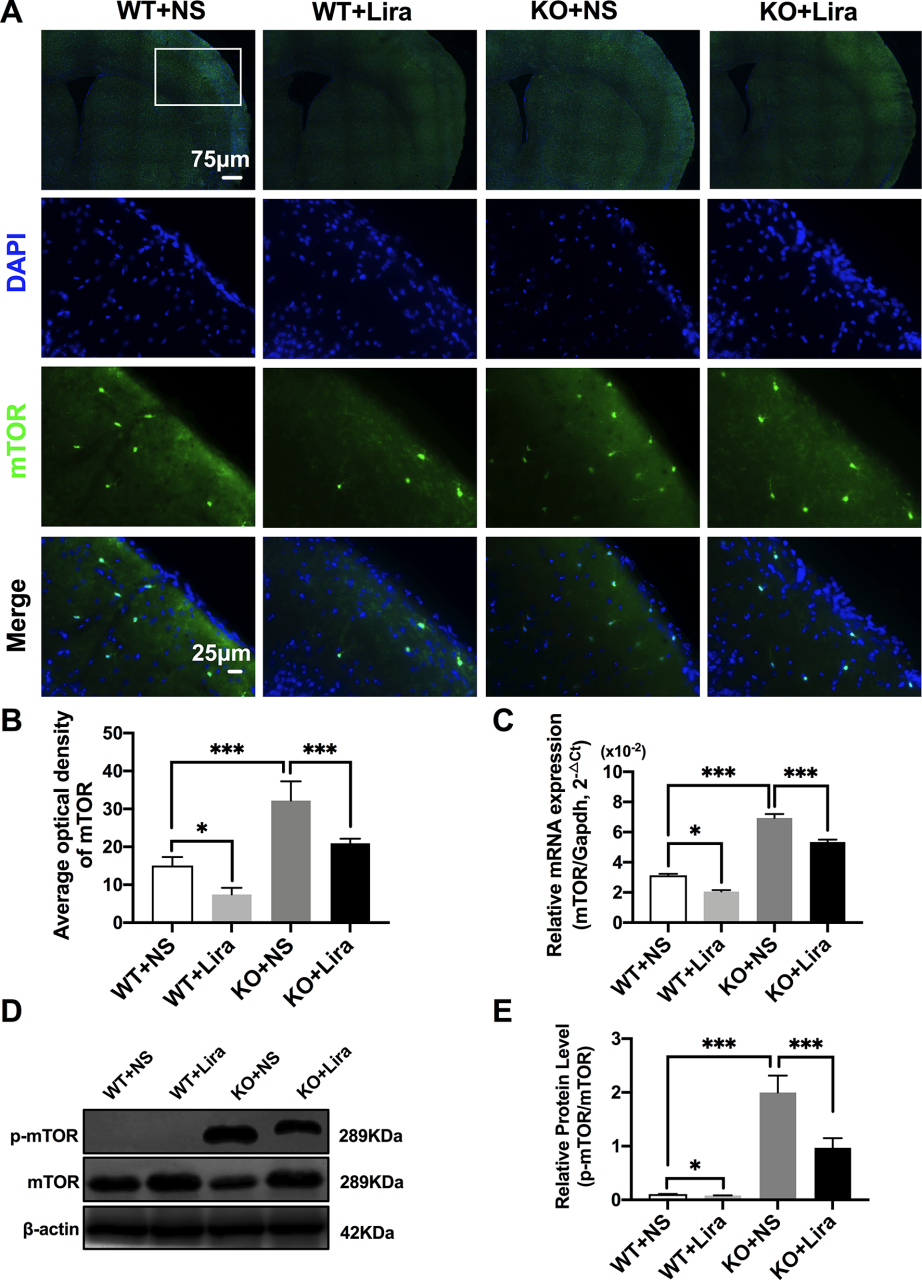
Liraglutide inhibited phosphorylation mTOR hyperactivation in *Scn1a* KO-induced epileptic mice. **(A)** Representative images of immunofluorescent staining with mTOR (green), and DAPI (blue) in the ipsilateral side of the cortex of the WT and *Scn1a* KO mice. Scale bar: 75 μm for the full-scale images and 25 μm for the magnified images. **(B)** Summary graph of the average optical density of mTOR fluorescent staining measured in images (A) demonstrating a decrease of mTOR in the cortex of *Scn1a* KO mice after liraglutide administration (N = 24 mice per group; *Student’s t-test, P* < 0.001). **(C)** RT-qPCR analysis of the mTOR mRNA expression from brains of wild-type (WT) and *Scn1a* KO heterozygote (KO) mice (N = 24 mice per group; *One-way ANOVA, Student’s t-test*). **(D)** Representative western blotting showing mTOR protein expression in brains of WT and *Scn1a* KO mice. **(E)** Summary graph of western blotting analysis demonstrating a significant reduction in the mTOR protein in brains of *Scn1a* KO as compared to WT mice after liraglutide administration (N = 24 mice per group; *Student’s t-test*, *P* < 0.001). The relative expression of SCN1A was normalized to reference controls *Gapdh* and β-actin in RT-qPCR and Western blotting, respectively. Data were presented as mean ± SD; *, *** represent *P* < 0.05, and *P* < 0.001, respectively. All experiments were performed in triplicate. WT, Wild-type; KO, *Scn1a* Knockout heterozygotes (F1); NS, Normal saline; Lira, Liraglutide.

Activation of the mTOR pathway has been reported to be related to epileptogenicity. Furthermore, mTOR hyperactivation was observed in genetic and acquired epilepsy syndromes (Citraro et al., 2016). The mTOR signaling pathway was examined in cortical neurons using immunofluorescence staining. The results revealed that mTOR fluorescence intensity was significantly enhanced in *Scn1a* KO mice compared with WT mice (WT+NS: 15.08 ± 2.24, KO+NS: 32.18 ± 5.07, *P* < 0.001, **Figures 4A, B**). However, treatment with Lira significantly reduced fluorescence intensity in both WT (WT+NS: 15.08 ± 2.24, WT+Lira: 7.41 ± 1.77, *P* < 0.05) and *Scn1a* KO + Lira groups (KO+NS: 32.18 ± 5.07, KO+Lira: 20.94 ± 1.19, *P* < 0.001, **Figures 4A, B**). RT-qPCR was used to evaluate mTOR mRNA expression in WT and *Scn1a* KO mice and the results were consistent with those from immunofluorescence staining (**Figure 4C**). We further measured the protein expression ratio of p-mTOR/mTOR in WT and *Scn1a* KO mice by western blotting. The p-mTOR/mTOR ratio was significantly higher in the brain of *Scn1a* KO mice compared to that of WT mice (WT+NS: 0.11 ± 0.01, KO+NS: 1.99 ± 0.31, *P* < 0.001, **Figures 4D, E**). Importantly, Lira treatment significantly decreased the expression ratio of the p-mTOR/mTOR in *Scn1a* KO mice (KO+NS: 1.99 ± 0.31, KO+Lira: 0.97 ± 0.18, *P* < 0.001, **Figures 4D, E**), indicating that Lira can inhibit the mTOR signaling pathway in *Scn1a* KO-induced epileptic mice.

### Liraglutide Reduces Apoptosis in the Brain of *Scn1a* KO-Induced Epileptic Mice

**Figure 5:**
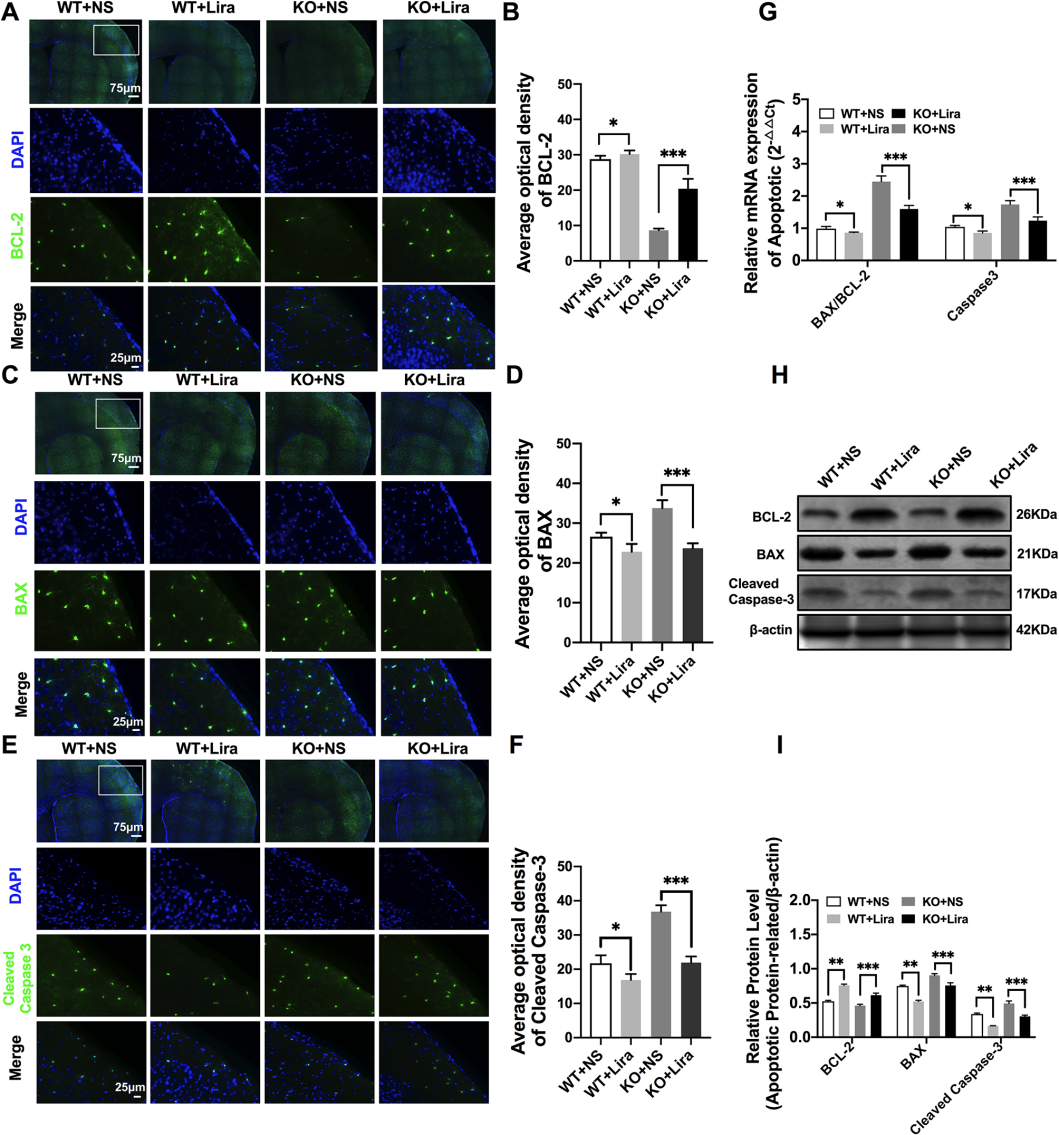
Liraglutide blocked apoptosis in *Scn1a* KO-induced epileptic mice. **(A, C, E)** Representative images of immunofluorescent staining with BCL-2/BAX/Cleaved Caspase-3 (green), and DAPI (blue) in the ipsilateral side of the cortex of the WT and *Scn1a* KO mice. Scale bar: 75 μm for the full-scale images and 25 μm for the magnified images. **(B, D, F)** Summary graph of the average optical density of BCL-2, BAX, and Cleaved Caspase-3 fluorescent staining measured in images (A, C, E) in the cortex of mice (N = 24 mice per group; *One-way ANOVA, Student’s t-test*). **(G)** RT-qPCR analysis of the BAX/BCL-2 and Caspase-3 expression from brains of wild-type (WT) and *Scn1a* KO heterozygote (KO) mice (N = 24 mice per group; *One-way ANOVA, Student’s t-test*). **(H)** Representative western blotting showing BAX/BCL-2 and Cleaved Caspase-3 protein expression in brains of WT and *Scn1a* KO mice. **(I)** Summary graph of western blotting analysis demonstrating a significant reduction in the apoptosis-related protein in brains of *Scn1a* KO as compared to WT mice after liraglutide administration (N = 24 mice per group; *Student’s t-test*, *P* < 0.001). The relative expression of apoptosis-related protein was normalized to reference controls *Gapdh* and β-actin in RT-qPCR and Western blotting, respectively. Data were presented as mean ± SD; *, **, *** represent *P* < 0.05, *P* < 0.01 and *P* < 0.001, respectively. All experiments were performed in triplicate. WT, Wild-type; KO, *Scn1a* Knockout heterozygotes (F1); NS, Normal saline; Lira, Liraglutide.

Apoptosis is a well-known and important feature of epilepsy-related diseases. Apoptosis was examined using immunofluorescence staining with regard to BCL-2, BAX, and cleaved caspase-3. The results revealed that BCL-2 fluorescence intensity was significantly enhanced in the *Scn1a* KO + Lira group compared with the *Scn1a* KO group (KO+NS: 8.66 ± 0.48, KO+Lira: 20.4 ± 2.78*, P* < 0.001, **Figures 5A, B**). Moreover, BAX and cleaved caspase-3 fluorescence intensity was significantly reduced in the *Scn1a* KO + Lira group compared with the *Scn1a* KO group (BAX, KO+NS: 33.72 ± 2.01, KO+Lira: 23.7 ± 1.22, *P* < 0.001; Cleaved caspase-3, KO+NS: 36.83 ± 1.84, KO+Lira: 21.94 ± 1.8, *P* < 0.001; **Figures 5C–F**). The mRNA and protein expression level of these apoptosis-related proteins was further evaluated using RT-qPCR and WB in the brain of both WT and *Scn1a* KO mice.

RT-qPCR revealed that the BAX/BCL-2 ratio and cleaved caspase-3 mRNA levels were significantly downregulated in the *Scn1a* KO + Lira group compared with the *Scn1a* KO group (BAX/BCL-2, KO+NS: 2.45 ± 0.17, KO+Lira: 1.59 ± 0.11, *P* < 0.001; Caspase-3, KO+NS: 1.74 ± 0.12, KO+Lira: 1.24 ± 0.11, *P* < 0.001; **Figure 5G**). Moreover, Lira treatment significantly decreased the ratio of BAX/BCL-2 and the expression of the apoptosis-related protein, cleaved caspase-3 in *Scn1a* KO mice (BCL-2, KO+NS: 0.46 ± 0.02, KO+Lira: 0.61 ± 0.02, *P* < 0.001; BAX, KO+NS: 0.90 ± 0.02, KO+Lira: 0.75 ± 0.04, *P* < 0.001; Cleaved caspase-3, KO+NS: 0.49 ± 0.04, KO+Lira: 0.30 ± 0.01, *P* < 0.001; **Figures 5H, I**). These data suggest that Lira administration can inhibit apoptosis in the brain of *Scn1a* KO mice.

### Liraglutide Promotes Proliferation and Reduces Apoptosis by Inhibiting mTOR Hyperactivation in *Scn1a* KO Cells

**Figure 6:**
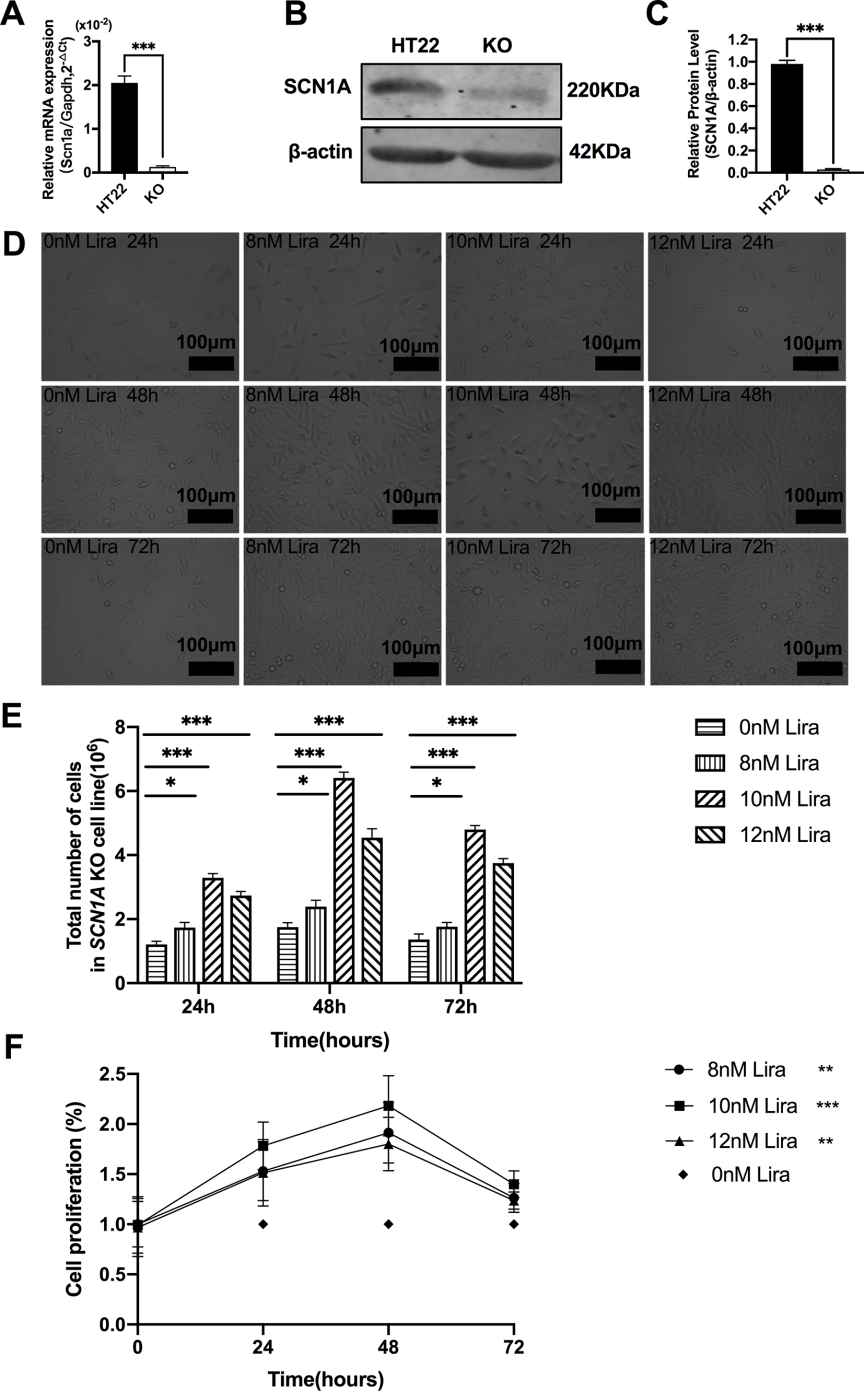
Liraglutide promoted the proliferation of *Scn1a* knockout HT22 cell in a time-and-dose relationship. **(A)** RT-qPCR analysis of the *Scn1a* mRNA expression from HT22 and *Scn1a* KO cell (*Student’s t-test*, *P* < 0.001). **(B)** Representative western blotting showing SCN1A protein expression in the HT22 and *Scn1a* KO cell. **(C)** Summary graph of western blotting analysis demonstrating a significant reduction in the SCN1A protein in *Scn1a* KO as compared to HT22 cell (*Student’s t-test*, *P* < 0.001). **(D)** Morphological changes of *Scn1a* KO cell in different time treated with different concentrations of liraglutide. **(E)** Total number of cells in *Scn1a* KO cell line (*Two-way ANOVA*). **(F)** Cell proliferation by CCK-8 (*Two-way ANOVA*). The relative expression of SCN1A was normalized to reference controls *Gapdh* and β-actin in RT-qPCR and Western blotting, respectively. Data were presented as mean ± SD; *, **, *** represent *P* < 0.05, *P* < 0.01 and *P* < 0.001, respectively. All experiments were performed in triplicate. WT, Wild-type; KO, *Scn1a* Knockout in HT22 cell; Lira, Liraglutide.

We examined the effect of Lira on the apoptosis and proliferation in a *Scn1a* knockout cell line model. Given the previous work in the authors’ laboratory (Shi et al., 2019), the *Scn1a* knockout in HT22 cell line was established using Crispr/cas 9 technology, and RT-qPCR and WB were used to verify the *Scn1a* KO cell line. As shown in **Figure 6A**, the level of SCN1A mRNA expression in the *Scn1a* KO cells was significantly lower compared with the HT22 control cells (HT22: 2.05 ± 0.16, *Scn1a* KO: 0.12 ± 0.02*, P* < 0.001, **Figure 6A**). In addition, the markedly decreased SCN1A protein expression level in *Scn1a* KO cells was measured by WB (HT22: 0.98 ± 0.03, *Scn1a* KO: 0.02 ± 0.01, *P* < 0.001, **Figures 6B, C**). The viability of *Scn1a* KO cells under different concentrations (8, 10, and 12 nM) of Lira was determined using the CCK-8 assay at 24, 48 and 72 h, respectively. The total number of *Scn1a* KO cells were measured using a cell counter (**Figures 6D, E**). Based on the results, Lira did not exhibit toxicity at lower doses (*P* > 0.05; **Figure 6F**) and hardly impacted cell viability at higher doses when its concentration was lower than or equal to 10 nM (**Figures 6D–F**); thus, 48 h was the appropriate time for drug treatment. Therefore, 10 nM Lira was used in subsequent experiments.

**Figure 7:**
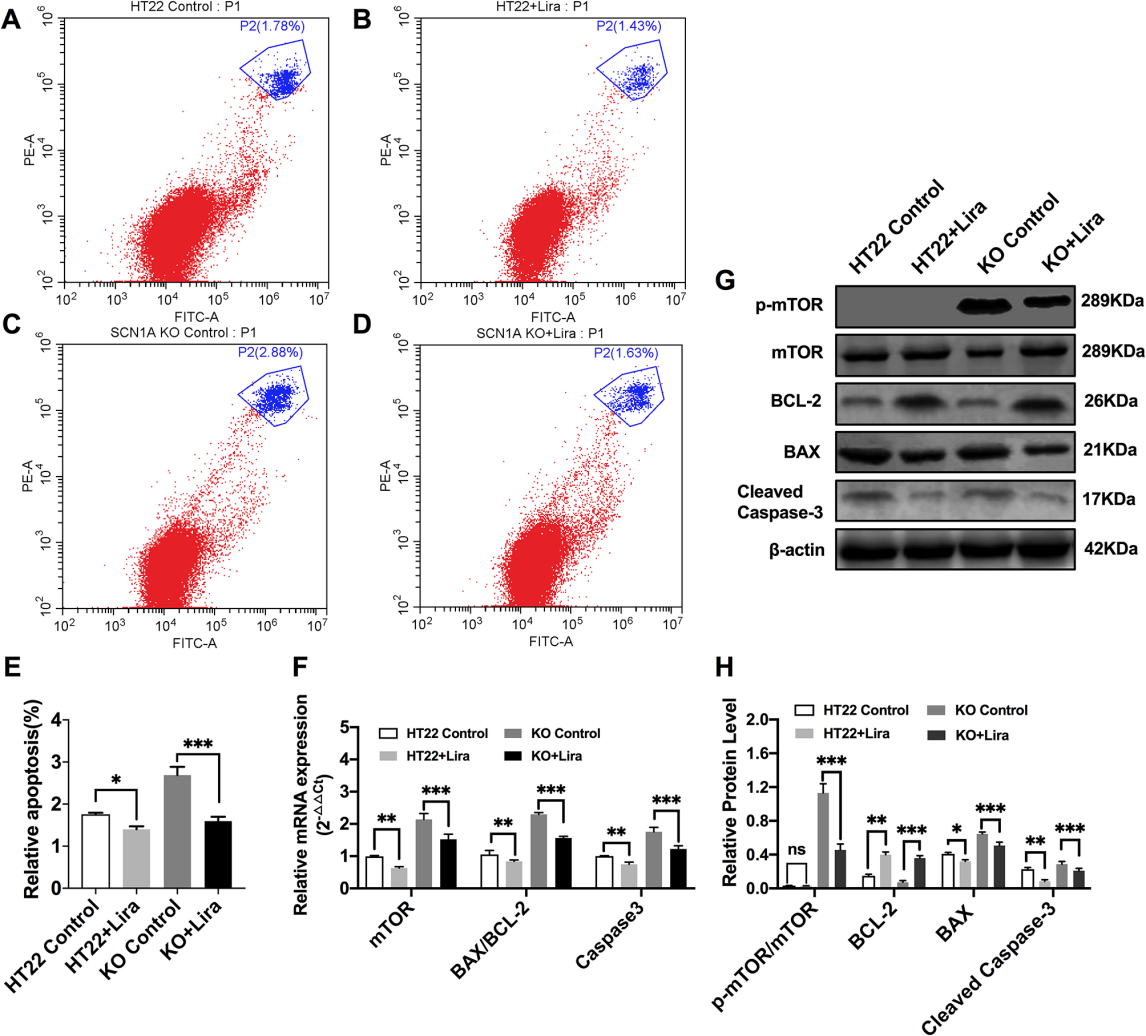
Liraglutide promoted proliferation and reduced apoptosis by inhibiting phosphorylation mTOR hyperactivation in *Scn1a* KO cell. **(A–D)** Annexin V-FITC/PI flow cytometry evaluating apoptosis in *Scn1a* KO cell model. **(E)** The percentage of induction of apoptosis in cell treated with the 10 nM Lira in comparison with the control group and 48 h of incubation (*One-way ANOVA*). **(F)** RT-qPCR analysis of the BAX/BCL-2, Caspase-3, and mTOR expression from HT22 and *Scn1a* KO cell (*One-way ANOVA, Student’s t-test*). **(G)** Representative western blotting showing BAX/BCL-2, Cleaved Caspase-3, and p-mTOR/mTOR protein expression in the HT22 and *Scn1a* KO cell. **(H)** Summary graph of western blotting analysis demonstrating a significant reduction in the apoptosis-related and mTOR protein in *Scn1a* KO as compared to HT22 cell after liraglutide administration (*Student’s t-test*, *P* < 0.001). The relative expression was normalized to reference controls *Gapdh* and β-actin in RT-qPCR and Western blotting, respectively. Data were presented as mean ± SD; *, **, *** represent *P* < 0.05, *P* < 0.01 and *P* < 0.001, respectively. All experiments were performed in triplicate. WT, Wild-type; KO, *Scn1a* Knockout in HT22 cell; Lira, Liraglutide.

Furthermore, Annexin V-FITC/PI flow cytometry was used to evaluate apoptosis in the *Scn1a* KO cell model, and revealed that the apoptotic rate of both HT22 control and *Scn1a* KO cells was significantly decreased after Lira treatment (HT22 control: 1.75 ± 0.03, HT22+Lira: 1.40 ± 0.07, *Scn1a* KO: 2.68 ± 0.19, *Scn1a* KO+lira: 1.59 ± 0.10, *P* < 0.001; **Figures 7A–E**). The expression level of apoptosis-related proteins was also evaluated using RT-qPCR and WB in both HT22 control and *Scn1a* KO cells. RT-qPCR revealed that the BAX/BCl-2 ratio and cleaved caspase-3 mRNA levels were significantly downregulated in the *Scn1a* KO cells treated with Lira (mRNA: BAX/BCL-2, *Scn1a* KO: 2.29 ± 0.05, *Scn1a* KO+Lira: 1.56 ± 0.05; Caspase-3, *Scn1a* KO: 1.75 ± 0.13, *Scn1a* KO+Lira: 1.22 ± 0.1; *P* < 0.001; **Figure 7F**). Moreover, Lira treatment significantly decrease the ratio of BAX/BCL-2 and the expression of the apoptosis-related protein, cleaved caspase-3 in *Scn1a* KO cells (protein: BCL-2, *Scn1a* KO: 0.07 ± 0.02, *Scn1a* KO+Lira: 0.36 ± 0.03; BAX, *Scn1a* KO: 0.65 ± 0.02, *Scn1a* KO+Lira: 0.51 ± 0.04; Cleaved Caspase-3, *Scn1a* KO: 0.28 ± 0.03, *Scn1a* KO+Lira: 0.21 ± 0.02; *P* < 0.001; **Figures 7G, H**).

We further evaluated whether mTOR activation was affected by Lira in both HT22 control and *Scn1a* KO cells. RT-qPCR was used to evaluate mTOR mRNA expression in HT22 and *Scn1a* KO cells. Compared with the *Scn1a* KO group, mTOR mRNA was significantly downregulated in the *Scn1a* KO + Lira group (*Scn1a* KO: 2.14 ± 0.18, *Scn1a* KO+Lira: 1.52 ± 0.16, *P* < 0.001, **Figure 7F**). Compared with the *Scn1a* KO group, the p-mTOR/mTOR protein ratio was significantly downregulated in the *Scn1a* KO + Lira group (*Scn1a* KO: 1.13 ± 0.11, *Scn1a* KO+Lira: 0.45 ± 0.07, *P* < 0.001, **Figures 7G, H**). These data suggest that Lira modulates *Scn1a* KO-induced apoptosis in TH22 cells *via* the mTOR signaling pathway *in vitro*.

## Discussion

The results of the present study demonstrate that Lira reduces seizure susceptibility, minimizes cognitive dysfunction, and inhibits apoptosis in neurons in *Scn1a* KO-induced epileptic mice and, moreover, decreases the levels of mTOR hyperactivation. Behavioral seizures in *Scn1a* KO-induced epilepsy models show that SCN1A deficiency increases susceptibility to seizures; however, treatment with Lira can reduce susceptibility and severity. In addition, *Scn1a* KO deficiency exacerbates the typical pathological manifestations of epilepsy in *Scn1a* KO mice. However, Lira intervention can ameliorate this pathological change. Neuroprotective effects were also observed in *Scn1a* KO HT22 cell model after Lira treatment. Hence, these findings suggest that the inhibition of apoptosis *via* inactivation of mTOR phosphorylation by Lira may partially contribute to its effects on epileptogenesis, seizure relief, and neuroprotection.

DS is a severe epileptic encephalopathy, most often resulting from *de novo SCN1A* mutations (Lorincz and Nusser, 2010), and typically begins in infancy with seizures provoked by fever, including status epilepticus cognitive impairment (Sugiura et al., 2012; Beck et al., 2019), and poor response to available antiepileptic drugs. Voltage-gated sodium channels are protein complexes consisting of one alpha subunit and one or more beta subunits to mediate action potentials in excitable tissues (Whitaker et al., 2001; Tsukamoto et al., 2017). The mammalian genome has nine homologs of sodium channel (SCN1A-SCN5A and SCN8A-SCN11A) encoding nine sodium channel alpha subunits (Nav1.1–Nav1.9), respectively. The alpha subunit is the major component of the sodium channel and is the functional unit. Nav1.1 channels are expressed in the cortex, hippocampus, cerebellum, and olfactory bulb (Escayg and Goldin, 2010; Lorincz and Nusser, 2010; Brackenbury and Isom, 2011). Scn1a^+/-^ heterozygous KO mice mimic features of DS and provide a potential screening platform to investigate novel therapeutics (Kalume et al., 2013; Miller et al., 2014; Mistry et al., 2014; Kang et al., 2019). We used the F1 heterozygous *Scn1a* KO mouse model, and the clinical presentation and identification were consistent with previous literature reports (Cheah et al., 2012; Miller et al., 2014; Mistry et al., 2014). *Scn1a* KO mice were injected with Lira for 14 days and its effects were evaluated. EEG, IntelliCage, and the open field task experiment has shown that Lira could reduce seizure susceptibility and cognitive dysfunction in *Scn1a* KO-induced epileptic mice. Recurrent seizures are often concomitant with neuronal loss in human patients and in animal models (Curia et al., 2014). Thus, we used HE and Nissl staining to observe neuronal death and degeneration and evaluate the effects of Lira in *Scn1a* KO-induced epileptic mice. HE and Nissl staining revealed that Lira alleviated neuronal damage following *Scn1a* KO-induced status epilepticus. Furthermore, IF results demonstrated that Lira increased the expression levels of *Scn1a* in *Scn1a* KO-induced epileptic mice, which was further supported by RT-qPCR and WB results. These findings suggest that Lira treatment delays the epileptic developmental process, reduces seizure susceptibility, alleviates neuronal damage, and minimizes behavior and cognitive deficits in *Scn1a* KO-induced epileptic mice.

Apoptosis is a type of the programmed cell death, and two major pathways can induce apoptosis, namely, the mitochondrial apoptotic pathway (intrinsic pathway) and the death receptor apoptotic pathway (extrinsic pathway) (O’Neill et al., 2016; Green, 2017; Wang et al., 2017). Increasing membrane permeability can mediate the release of pro-apoptotic proteins from mitochondria to activate the intrinsic apoptotic pathway controlled by the Bcl-2 superfamily. Combined transmembrane death receptors with ligands, such as Fas ligand (FasL) and tumor necrosis factor (TNF), can activate the extrinsic pathway. Both pathways converge at the level of effector caspase, and then activate caspase-3 and caspase-7 to induce apoptosis (Rongvaux et al., 2014; Kayagaki et al., 2015). Seizures can cause apoptosis, which in turn can promote the progression of epilepsy (Engel et al., 2010; Fu et al., 2010). Considering the effects of apoptotic mechanisms, the results are not surprising, and apoptosis induction may be the most common target for epilepsy treatment. In our experiments, we used the expression levels of cleaved caspase-3, BCL-2 associated X protein (BAX), and B cell lymphoma 2 (BCL-2) to reflect the extent of apoptosis. We demonstrated that the administration of Lira downregulated the expression of cleaved caspase-3 and the ratio of BAX to BCL-2 in *Scn1a* KO models. Therefore, Lira can reduce neural apoptosis in *Scn1a* KO-induced epileptic mice potentially *via* inhibition of BAX/BCL-2 signaling.

mTOR is a serine/threonine kinase, divided into two multimeric active forms: rapamycin-sensitive mTOR complex (mTORC1) and mTOR complex 2 (mTORC2) (Lipton and Sahin, 2014; Citraro et al., 2016). The core molecules of mTORC1 and mTORC2 are, respectively, the regulatory-associated protein of mTOR (Raptor) and (Rictor). mTORC1 is sensitive to nutrients and regulates protein synthesis and cell growth through the downstream molecules 4E-BP1 and S6K. mTORC2 is regulated *via* PI3K and growth factor signaling and is responsive to growth factor signaling by phosphorylating the C-terminal hydrophobic motif of some AGC kinases such as Akt and SGK (Linke et al., 2017; Hua et al., 2019; Yang et al., 2019). mTOR is a key regulatory node in growth-factor signaling across evolution. Its importance in a wide array of physiological processes, including insulin signaling, cell growth, immunity, and brain development, has been confirmed in animal models and in humans (Goncalves et al., 2018). Moreover, mTOR hyperactivation has been observed in genetic and acquired epilepsy syndromes (Liu et al., 2014). In the present study, we found that Lira inhibited the hyperactivation of mTOR in *Scn1a* KO mice. Therefore, Lira can exert neuroprotective effects in *Scn1a* KO-induced epileptic mice by inhibiting the mTOR signaling pathway.

GLP-1 is an incretin hormone used to treat diabetes mellitus. GLP-1R are not only distributed in intestinal L cells, but also in the central nervous system (CNS) (Richards et al., 2014; Katsurada and Yada, 2016). We hypothesize that this widespread distribution of GLP-1R may be the anatomical basis for GLP-1 analogues to exert neuroprotective effects. It has been reported in some research that GLP-1 analogues demonstrate neuroprotective effects in acute and chronic epileptic mouse models. GLP-1 analogs may exert anti-epileptic effects through apoptotic pathways in epileptic mouse models (Koshal and Kumar, 2016; Hussein et al., 2019; Wen et al., 2019). Moreover, the mTOR signaling pathway has been shown to be involved in regulating neuronal function, proliferation, apoptosis, and other cellular processes associated with epileptogenesis. Studies have shown that the activity of the mTOR signaling pathway is abnormally increased in epilepsy models, and that GLP-1 analogs can regulate mTOR expression *via* the AMPK pathway (Hurtado-Carneiro et al., 2012; Kong et al., 2018). In our experiments, we found that apoptosis and mTOR expression were increased in *Scn1a* KO mice and cell models. However, this effect can be reversed after intervention with GLP-1 analogue.

In summary, the current study has demonstrated that Lira can significantly inhibit hyperactivation of the mTOR signaling pathway and reduce neural apoptosis in *Scn1a* KO mice and cell models. Although experimental animal and cell models have been used for elucidating these correlatives, further experimental research and rigorous clinical investigations should be conducted in human-based, clinical studies.

## Data Availability Statement

All datasets generated for this study are included in the article/**Supplementary Material**.

## Ethics Statement

The mice were handled according to the guidelines prescribed by the Institutional Animal Care and Use Committee of Ningxia Medical University (IACUC Animal Use Certificate No.: SCXK (Ning) 2015 – 0001).

## Author Contributions

Study design: TS, FW, ZJ. Experiment implementation: SL, SR, WH. Paper writing: SL, YZ, ZJ. Data analysis: SL, ZJ, YZ, SR, KS, LX, XL, DW, and ND. All authors read and approved the final manuscript.

## Funding

This study was supported by the National Natural Science Foundation of China (NSFC) (NO. 81660226), the key R & D project of the autonomous region (No. 2018BFG02007), and the Ningxia Hui Autonomous Region “13th Five-Year Plan” Major Science and Technology Projects (Ningxia Brain Project) (No. 2016BZ07).

## Conflict of Interest

The authors declare that the research was conducted in the absence of any commercial or financial relationships that could be construed as a potential conflict of interest.

## Abbreviations

SCN1A, NaV1.1 sodium channel; CNS, central nervous system; DS, Dravet syndrome; GLP-1, glucagon-like peptide-1; GLP-1R, glucagon-like peptide-1 receptor; HE, hematoxylin and eosin; IF, immunofluorescence; KO, knockout; Lira, liraglutide; mTOR, mammalian target of rapamycin; PCR, polymerase chain reaction; RT-qPCR, Reverse transcription-quantitative polymerase chain reaction; WB, western blotting; WT, wild type.
