# Supplementary figures and images for: The Glucagon-Like Peptide-1 Analogue Liraglutide Reduces Seizures Susceptibility, Cognition Dysfunction and Neuronal Apoptosis in a Mouse Model of Dravet Syndrome
Source: Front Pharmacol. 2020 Feb 28;11:136. doi: 10.3389/fphar.2020.00136 (PMC7059191; doi:10.3389/fphar.2020.00136)

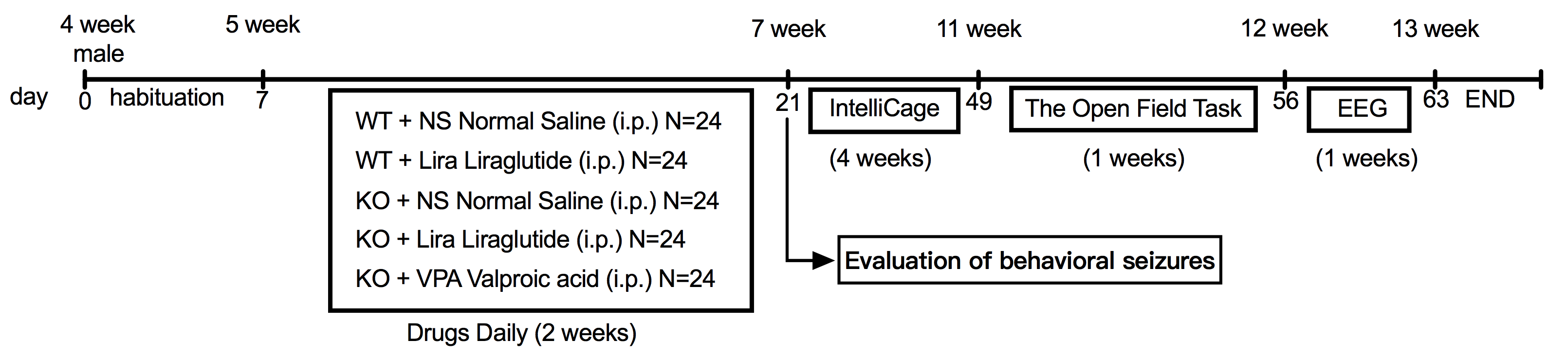

Supplement: Supplementary file 1 [file Image_1.tiff]

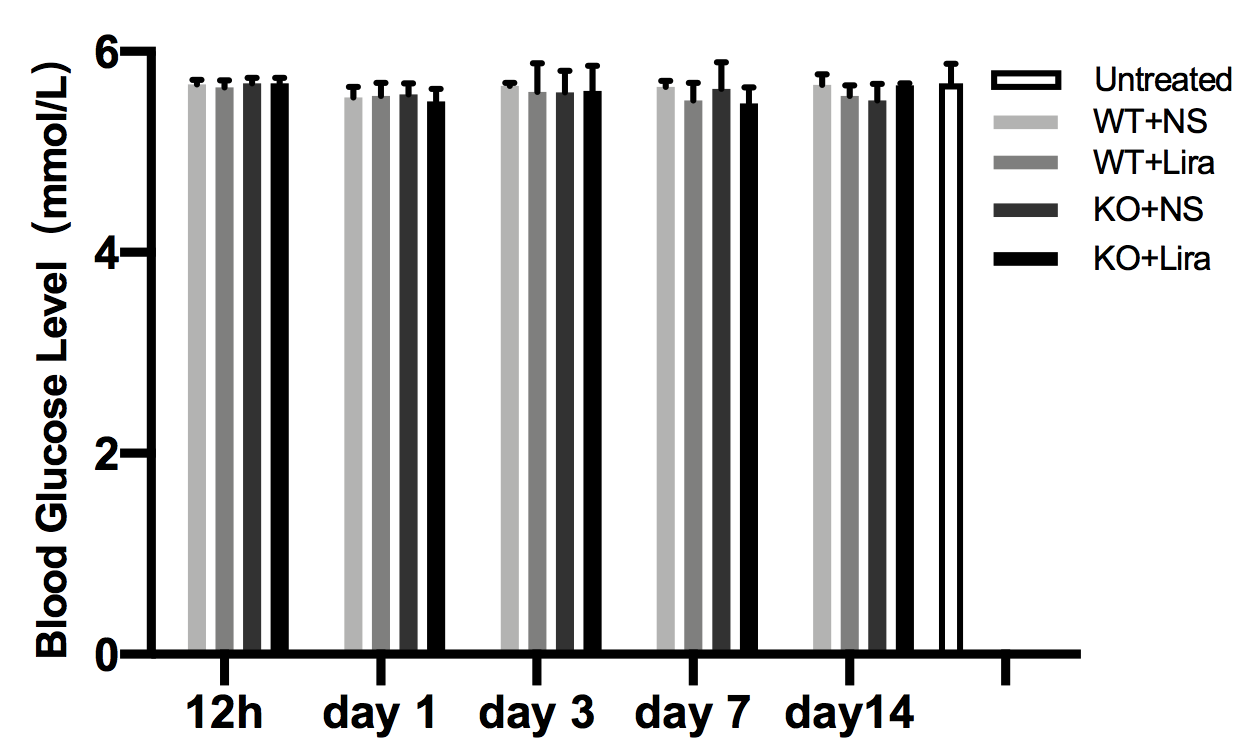

Supplement: Supplementary file 2 [file Image_2.tiff]
