# Supplementary material for: The Glucagon-Like Peptide-1 Analogue Liraglutide Reduces Seizures Susceptibility, Cognition Dysfunction and Neuronal Apoptosis in a Mouse Model of Dravet Syndrome
Source: Front Pharmacol. 2020 Feb 28;11:136. doi: 10.3389/fphar.2020.00136 (PMC7059191; doi:10.3389/fphar.2020.00136)
Supplement: Supplementary file 3 [file Table_1.docx]

**Supplementary Table1**

Primers used for reverse transcription-quantitative polymerase chain reaction

| Genes | Primer/probe | Primer /probe sequences | |
| --- | --- | --- | --- |
| GAPDH  mTOR  Caspase 3  BAX  BCL-2  SCN1A | Forward primer  Reverse Primer  Forward primer  Reverse Primer  Forward primer  Reverse Primer  Forward primer  Reverse Primer  Forward primer  Reverse Primer  Forward prime  Reverse Primer | 5’-TGTTGCCATCAATGACCCCTT-3’  5’-CTCCACGACGTACTCAGCG-3’  5’-TGACAATGGCATCGTGCCTCC-3’  5’-GGCGTATTCCAACACCCCAG-3’  5’-GAGCTGGACTGTGGCATTGAGAC-3’  5’-TGCGCGTACAGCTTCAGCATG-3’  5’-CGTGAGCGGCTGCTTGTCTG-3’  5’-ATGGTGAGCGAGGCGGTGAG-3’  5’-ACGGTGGTGGAGGAACTCTTCAG-3’  5’-AGATGCCGGTTCAGGTACTCAGTC-3’  5’-TCCTGGAGGGTGTTTTAGATGC-3’  5’-AAAGATTTTCCCAGAAGTCCTGAG-3’ |  |
